# ‘I became more aware of my actions’—A qualitative longitudinal study of a health psychological group intervention for patients with myalgic encephalomyelitis/chronic fatigue syndrome

**DOI:** 10.1111/hex.13833

**Published:** 2023-08-01

**Authors:** Meeri Keurulainen, Juha Holma, Elina Wallenius, Mikko Pänkäläinen, Jukka Hintikka, Markku Partinen

**Affiliations:** ^1^ Department of Psychology University of Jyväskylä Jyväskylä Finland; ^2^ Wellbeing Services County of Päijät‐Häme, General Hospital Psychiatry Outpatient Clinic Lahti Finland; ^3^ Faculty of Medicine and Health Technology University of Tampere Tampere Finland; ^4^ Faculty of Medicine University of Helsinki Helsinki Finland; ^5^ Terveystalo Helsinki Sleep Clinic Helsinki Finland

**Keywords:** chronic fatigue, cognitive behavioural therapy, health psychology, illness management, ME/CFS, qualitative longitudinal study

## Abstract

**Objectives:**

To explore myalgic encephalomyelitis/chronic fatigue syndrome (ME/CFS) patients' experiences of a health psychological group intervention and its usefulness, non‐usefulness or harmfulness for illness management and adjustment.

**Design:**

A qualitative longitudinal study using inductive content analysis.

**Methods:**

Semistructured interviews were conducted with 10 adults. Interviews were conducted before the 16‐week intervention, immediately after its completion, and at 3 months after completion.

**Results:**

Participants reported that the intervention was useful and not harmful. The model improved their ability to cope with ME/CFS by providing them with useful information about the illness along with peer support and professional guidance. Participants reported improved illness management and adjustment, which they perceived as an outcome of achieving new ways of thinking, feeling and acting.

**Conclusions:**

Participants viewed the health psychological approach to group intervention as meeting their needs. To achieve better illness management and adjustment, more consideration should be given to supportive interactional processes with peers and healthcare professionals.

**Patient or Public Contribution:**

The intervention was developed to meet patients' needs of finding ways to manage their illness. The research team consulted eight patients with ME/CFS and three clinical centres working with ME/CFS treatment and rehabilitation at the intervention planning stage. Their comments influenced the planning and content of the intervention as well as ethical issues that should be considered, such as potential harm to participants. All participants were informed about the theoretical foundations of the study and the principles guiding the intervention. Participants were not involved in the data analysis.

**Clinical Trial Registration:**

NCT04151693

## 1 INTRODUCTION

ME/CFS (myalgic encephalomyelitis/chronic fatigue syndrome; G93.3) is a long‐lasting and difficult‐to‐diagnose illness, characterised by long‐term fatigue and malaise, that cannot be explained by any other physical or psychiatric illness. Physical or mental stress worsens fatigue and malaise, and rest does not improve the patient's condition.^1^, ^2^, ^3^, ^4^, ^5^ There is no one cure fits all for ME/CFS. However, functional capacity and dysfunction of the autonomic nervous system can be improved and maintained with nondrug rehabilitation and symptomatic drug treatments. Moreover, secondary diseases can be treated.^1^, ^2^, ^3^, ^4^, ^5^ An individually planned rest and exercise plan and appropriately paced activities are also recommended.^4^, ^5^ It is essential that sufferers learn to listen to their bodies, understand its limits and rehabilitate on its terms. Bed rest/rest alone or progressing too quickly can worsen symptoms.

The results of studies on the effectiveness of individual‐ and group‐level cognitive behavioural therapy models (CBTs) in treating ME/CFS have been conflicting.^1^, ^2^, ^3^, ^4^, ^5^, ^6^, ^7^, ^8^, ^9^, ^10^ Some studies have found CBTs to be effective and others have found their use to be harmful. Historically, ME/CFS has been described by nine different criteria, with differing emphases on symptoms and differing aetiological assumptions about whether the disease is physical or psychiatric. According to those favouring a psychiatric or psychological aetiology, ME/CFS is maintained by unhelpful beliefs about exercise, whereas reconditioning the body can lead to recovery from the illness.^6^ Thus, CBT therapies were earlier researched with the aim of finding a cure for the illness. Nowadays, the role of CBT in the treatment of ME/CFS is seen differently, that is, not as a cure for the illness but as an aid to illness management, reducing illness distress and promoting adjustment.^4^ In CBT‐based models, the number of sessions and themes are determined in advance. Frequent themes are identifying negative thoughts, practising new skills, goal setting, problem solving and self‐monitoring. The goal of interventions based on accept and commitment therapy is to promote a life in line with the client's values and psychological flexibility through the activation of awareness and acceptance skills and value‐oriented behaviour.^11^, ^12^ Thus, psychological support for patients with ME/CFS has shifted away from a diagnosis‐oriented approach to a more holistic, health psychological approach.

Health psychological approaches focus on overall health on the assumption that biological, psychological, and social factors influence each other in all stages of sickness and health. Illnesses can be treated and managed not only biomedically but also with biopsychosocial instruments. The focus is also on health‐related behaviour and resources and processes, such as illness‐related distress and adaptation, where the aim is constructive adaptation to the circumstances of one's life and maintaining and improving one's functional capacity as much as possible.^13^, ^14^, ^15^, ^16^, ^17^, ^18^, ^19^, ^20^, ^21^, ^22^, ^23^, ^24^, ^25^, ^26^, ^27^, ^28^

In society and especially in the health care system, ME/CFS patients continue to face an illness that is a misunderstood and its existence even denied.^6^ Desired and needs‐meeting support, such as information about the diagnosis, respect, empathy, and support from healthcare workers, and social benefits, tools for rehabilitation, illness management and adjustment, are lacking, leading patients to turn to self‐care and resources they find in patient networks.^29^ Negative social representations linked to the lack of a medical cure likely contribute to reinforcing peer support versus physician support.^30^

Although ME/CFS patients' needs are known in healthcare, tools for supporting the illness continue to be lacking. Moreover, ME/CFS has a long, contradictory, and stigmatised history, which even today affects the attitudes of nursing staff and hence patient care and treatment. To remedy this situation, ME/CFS studies and interventions should focus more on the role of interactive processes, such as encounters with healthcare workers, or facilitators and barriers to adaptation and rehabilitation processes in the social environment. Hareide et al.^31^ found that besides symptom intensity, information disseminated by health care professionals and identification with the diagnosis seemed to affect ME/CFS patients' coping behaviour. They concluded that multiple perspectives, such as individual differences, developmental factors and a relational focus, especially medical encounters, should be included in future research. Multiple perspectives and patients' needs should also be considered in intervention planning.

This study explored the experiences of a CBT‐based and tailored health psychological group intervention for patients with ME/CFS. We addressed four research questions:

1. What were the participant's goals, and were they attained?
2. Did the participants experience the intervention as useful, and if so, in what way(s)?
3. Did the participants experience the intervention as harmful, and if so, in what way(s)?
4. Did the participants experience the intervention as not useful, and if so, in what ways?

## 2 METHODS

### 2.1 Procedure

This study is part of a multidisciplinary research project aimed at developing and evaluating a health psychological group intervention tailored to patients with ME/CFS that is being conducted in a specialised care unit at Päijät‐Häme Central Hospital in Finland. The project was ethically approved in June 2019 and permission to implement it was granted in September 2019. The data collection began in October 2019.

Participation in the study was voluntary and all participants gave their informed written consent. Participation required a clinically confirmed diagnosis (G93.3) and meeting the consensus criteria of both the Institute of Medicine (IOM) and the Myalgic Encephalomyelitis of Canada (ICC 2011). Exclusion criteria were other diseases (assessed by laboratory tests), psychiatric diseases and severe personality disorders (assessed by the Structured Clinical Intervew for DSM). SOMNOtouch NIBPTM (Somnomedics GmBh) continuous pulse‐to‐pulse blood measurement and an interview (capability, motivation and goal setting) with a psychologist were also implemented to assess suitability for participation in the study.

The present study explored participants' experiences of the usefulness and harmfulness of a group intervention for ME/CFS patients. The intervention is a CBT‐based, tailored programme focused on improving health‐related behaviour. For this reason, we included the term health psychological in the name of the intervention, as it best describes its theoretical background.

**Figure 1:**
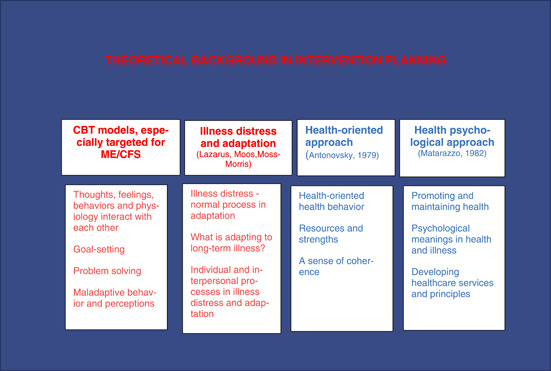
Theoretical background in intervention planning.

**Figure 2:**
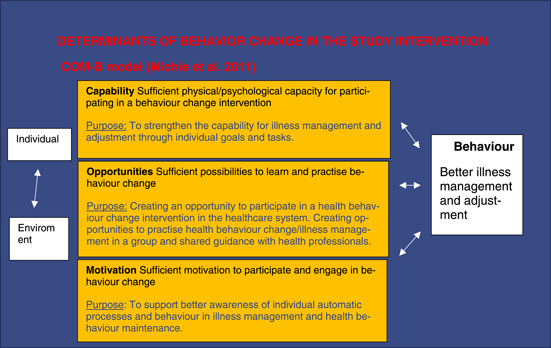
Determinants of behaviour change in the study intervention.

**Figure 3:**
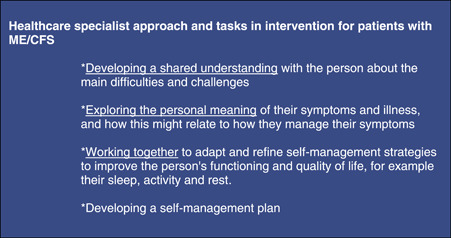
Healthcare specialist approach and tasks in the study intervention.

The research intervention was developed and implemented by the research group, mostly by the first and third authors. The development process began with literature research^1^, ^2^, ^3^, ^6^, ^7^, ^8^, ^9^, ^13^, ^14^, ^15^, ^16^, ^17^, ^18^, ^19^, ^20^, ^21^, ^22^, ^23^, ^24^, ^25^, ^26^, ^27^, ^28^, ^29^ to establish the theoretical basis for the intervention (Figure 1). We then defined the required behavioural change using the COM‐B model^32^ (Figure 2), created the first version of the intervention contents, and tested its novel results in clinical practice. Before it was implemented, the final intervention was evaluated by two patient groups (*n* = 8). Other experts in three clinical centres conducting research on ME/CFS treatment and rehabilitation (Mayo Clinic, Rochester, MN, USA; TAYS Tampere University Hospital, Finland; HUS Helsinki University Hospital, Finland) were consulted. The approach of the healthcare specialists guiding the intervention, which was defined in 2019, corresponded to the contents of NICE recommendations subsequently published in 2021 (Figure 3).

The contents of the research intervention (Table 1) was a combination of patients' wishes, consensus recommendations and the outcomes of ME/CFS‐related studies. The intervention topics included scientific knowledge related to ME/CFS, adaptation, stress management and working with one's thoughts. Patients' wishes, an experimental specialist, and a person who has the same illness and has been educated to share their experience of it, were included in two group intervention sessions (2 and 7) in 2022 aimed at inspiring the participants to find their own ways to manage their illness.

**Table 1:** Intervention content and topics.

Theme of session	Topics	Focus
1. What is ME/CFS?	Latest literature and theories Basics of rehabilitation strategies Goal‐setting check	Information, ME/CFS‐specific and general tools
2. Pacing and symptom management	Principles of pacing Week and daily Schedule Stress management *Experience specialist included	Information, ME/CFS‐specific and general tools
3. Better understanding of body signals	Sleep, nutrition, exercise Understanding alternative states of alertness	Body awareness
4. The illness crisis and adaptation to changes in functioning	What psychological adaption means Adaptation is not giving up but making a new plan How to handle my emotions My values in life	Emotional process
5. Thoughts, emotions, body, and action‐interaction	Basic understanding of human processing, neuroplasticity, and learning	Thought process
6. Finding alternative ways to think, identifying own maladaptive thought patterns	Thoughts reframe how we see the world Why it is important to focus on good Own maladaptive thoughts Finding new ways of reframing thoughts	Information, tools, body awareness, emotional process and thought process integrated
7. How do I see myself and others?	How I see myself Body image, sexuality, personality What I wish from others Communicating with others *Experience specialist included	Information, tools, body awareness, emotional process and thought process integrated
8. My plan	Hope and tools for moving forward Resilience and sense of coherence My goals—did I reach them? My tools and plan	Information, tools, body awareness, emotional process and thought process integrated
9. Follow up meeting‐ what's up after 3 months	What's up? How to move on from the present My goals for the future	Information, tools, body awareness, emotional process and thought process integrated

### 2.2 Participants

Participants were 10 patients, nine women and one man, drawn from research groups implemented in 2019 and 2022. Participants were originally recruited from units in different fields of special care, such as rheumatology and neurology, that sought to meet patients' need for managing their symptoms and adjusting to their illness. The participants had been diagnosed 1–3 years earlier and on average had experienced symptoms for approximately 2–5 years before attending the intervention. Their illness severity was classified, using the ICC 2011 criteria, as mild (8/10) and moderate to severe (2/10). Their mean age was 41 years and median 44 years (range 20–59). The participants were from the occupational fields of education, social and healthcare, trade, and information technology. All were white, and none had previously experienced an intervention of this kind. Five participants were in both the 2019 and 2022 groups. The intervention comprised eight 2‐h sessions over 16 weeks and one follow up‐meeting at 3 months postintervention. The same individuals were present in the same sessions, and thus got to know one another and share their experiences of managing their illness.

### 2.3 Data analysis and quality assurance

The data consisted of interviews conducted before the 16‐week intervention, immediately afterwards, and at 3 months after intervention end. The interviews were half‐structured. Participants were asked about their functioning, motivation for the intervention, experiences of the intervention and in the second and third interview, what they considered to be its benefits and harms (Supporting Information: Appendix 1). The first author conducted all the interviews and delivered the intervention.

The research secretary transcribed the interview talk verbatim. The average length of the first interview was 50 min and that of the second and third interviews 34 min. The average length of transcriptions was 15 pages, and the total 437 pages. A qualitative longitudinal approach^33^ was chosen as no previous intervention studies on this topic existed, and we were interested in participants' experiences of this specific intervention. Data were analysed using inductive content analysis.^34^ The researcher proceeded in analysis by following steps: preparation, organising and reporting. First, the unit of analysis was chosen. Meaning was selected as the unit of analysis. Next, the data were read multiple times and open‐coded. Three main themes relevant to the research questions were identified. A separate file was created for each main theme. The main themes were then subcategorised and divided into useful, harmful, and nonuseful experiences. The frequencies of the main themes and their subgroups were also calculated and tabulated. Samples representing the most common and interesting findings in the material were then selected from the data in the categories and subcategories. Unusual and research‐relevant findings were included to enrich the samples. Finally, key findings and their meanings were interpreted and incorporated into the manuscript.

All the participants' interviews were included in the data, although it was noticed by the sixth interview that the material was starting to repeat itself. However, because we wanted to ensure that the final categories represented the experiences of all the participants, the analysis of the data was not stopped at the point of saturation, and thus all the interviews were included.

Qualitative research guidelines^35^ were followed to enhance the reliability of the study. A research diary was kept by the first author. Coded transcripts and memos of different themes and subcategories were shared with the second, fifth and sixth co‐author. These co‐authors addressed comments and requests for elaboration to the first author. All comments were discussed and incorporated into the analysis. The research intervention was implemented by the third and fourth co‐author with no involvement from the second, fifth and sixth co‐authors. The first author was responsible for collecting the data and writing the first version of the manuscript. All authors participated in either the data collection and/or writing of the final version of the manuscript.

## 3 RESULTS

Table 2 presents the main findings. Of the 10 participants, six reported reaching all their goals and four partially reaching their goals. These four participants reported achieving some but not all their goals or improving in many.

My goals were fulfilled **partly. I got off to a good start**. In the group I learned why those breathing exercises really work but I must work more to use them properly. (Bellamy)

**Table 2:** Participants and main findings.

Particpiant	Age	Illness severity (ICC classification)	Educational background	Occupational status before intervention	Occupational status after intervention	Occupational status follow‐up (3 months)	Goals for the intervention	Experience of goals achieved second and third interview	Experienced change in symptoms from first interview to final interview	Experienced change in symptoms form second interview to third interview	Experienced intervention usefulness second and third interview	Suggestions for developing the intervention second and third interview	Own goals for the future second and third interview
1	20–25	Moderate to severe	Elementary school	Unemployment	Unemployment	Unemployment	Peer support	Yes	Mild worsened cognitive functioning, illness fluctuation.	No changes in physical or cognitive improvement, better emotional balance	Useful	Family support and Social benefit specialist	Adjusting and life quality
2	50–55	Mild	Vocational education	Full‐time worker	Full‐time worker	Full‐time worker	Pacing ability to continue working	Yes	Mild physical and cognitive improvement	Mild physical improvement	Useful	Family support	Perfectionistic thoughts, pacing, ability to continue working, exercise
3	50–55	Mild	MA	Full‐time worker	Full‐time worker	Part‐time worker, Student	Peer support	Yes	Mild physical and cognitive improvement	Mild physical improvement	Useful	Family support and nutrition counselling	Better overall functioning, working with thought patterns
4	20–25	Mild	University student	Student	Student	Student	Peer support, work with negative thoughts	Partly	Mild cognitive improvement	No changes from second interview	Useful	Family support	Negative thoughts, self‐image, pacing, ability to continue working, exercise
5	40–45	Mild	BA	Unemployment	Work‐trial	Part‐time worker	Peer support, work with negative thoughts	Partly	Mild physical and cognitive improvement, better emotional balance	Mild physical improvement	Useful	Family support	Part‐time job and studies, emotional balance, relaxing skills, work with cognitive thoughts
6	35–40	Mild	BA	Part‐time worker	Full‐time worker	Full‐time worker and student	Peer support, pacing, work with negative thoughts	Yes	Mild physical, emotional and cognitive improvement	Mild physical, emotional and cognitive improvement	Useful	Family support, working more with negative thoughts	Exercise, nutrition, self‐image, negative thoughts
7	55–60	Mild	MA	Part‐time worker	Part‐time worker	Part‐time worker	Pacing, relaxing, Ability to continue working	Partly	No change in physical, emotional or cognitive functioning	No change in physical, emotional or cognitive functioning	Useful	More scientific research and utilisation of health technology	Relaxing, pacing, Ability to continue working and exercise, thought patterns
8	50–55	Mild	BA	Unemployment	Work trial	Work trial	Peer support, relaxation, pacing	Yes	Mild emotional, physical and cognitive improvement	Mild emotional, physical and cognitive improvement	Useful	More working with negative thoughts	Relaxing, pacing, ability to continue working and exercise, working with negative thoughts
9	35–40	Moderate to severe	High school	Rehabilitation support	Rehabilitation support	Rehabilitation support	Peer support, relaxation, pacing	Partly	Mild emotional and physiological improvement	Mild worsened cognitive and physical functioning	Useful	Family support	Better balance in everyday life, pacing, nutrition, working with thought patterns
10	30–35	Mild	Vocational education	Unemployment	Work trial	Work trial	Peer support, relaxation, pacing	Yes	Mild physical and cognitive improvement	No change in physical, emotional or cognitive functioning	Useful	‐	Ability to continue working and exercise, working with negative thoughts

In the final interview, 8 of the 10 reported experiencing mild physical or cognitive improvement, that is, less experience of brain fog and/or increased capacity for preferred physical activities, which they attributed to better symptom management.

Through the process, **I learned to respect my limits** better and stop overdoing things all the time. Now I feel more balanced…and I've noticed that I tolerate stress better. My thinking is clearer, and I can go to the gym for short periods of time. (Hannah)

One of the 10 reported mildly worsened cognitive functioning but regarded it as a typical fluctuation in symptoms. In the follow‐up interview, a second participant also reported worsened functioning and attributed this to exceeding their personal stress tolerance. In the second and third interviews, nine participants named working with thought patterns as a future goal. The most wished‐for change was to include support for the family members in the intervention protocol.

The most valuable thing **would be to include support for the family**. Because this is also stressful for them. And they have no clue what to do. (Bellamy)

In the final and follow‐up interviews, all the participants reported the intervention as useful and none found it harmful. Some issues that they felt merited consideration we will present later in this section.

Through researchers' interpretation, three main categories were identified in the data: *information*, *peer support*, and *shared guidance in personal processes*. These categories, subcategories and their frequencies are presented in Table 3. The most frequent category was shared guidance in personal processes. Peer support was also found beneficial. Information was the category based on which the intervention was experienced as beneficial or not all that useful. Most typically, if the participant had not found it useful, it was because the experimental condition did not allow optimal cognitive functioning or the participant had already obtained the information elsewhere. Next, we present results by main and subcategories.

**Table 3:** Data categories and analysis units in final and follow‐up interviews.

Main category	Subcategory	Benefit	No benefit/useful for participant	Harmful	All
*Information*	Information acquired in the group	28	17		45
*Information*	Workbook	29	21		50
*Peer Support*	Getting hope and perspective from other sufferers	20	1		21
*Peer support*	Shared experiences	20	2		22
*Peer support*	Practical tools from others	32	9		41
*Shared guidance in personal processes*	Experiencing the safe solving of problems together	25	4		29
*Shared guidance in personal processes*	Growing self‐awareness	63	4		67
		217	58		**275**

### 3.1 Information

#### 3.1.1 Information acquired in the group

The participants reported that acquiring information about their illness, stress, and psychological adjustment was an important component of the intervention. Such information enabled participants to start reflecting on their personal experiences of their illness and individual ways of managing it.

ME/CFS‐based illness‐management theories, such as pacing and stress tolerance were found to be important topics and a tool for a behavioural change:

**In a group**, yes, I find our discussions useful, specially themes with **pacing** and **stress tolerance**?

Interviewer: ‘Yes…’

… also, psychological stress as well as physical, needs to be noticed, everything effects your daily resources and how you use them, **and you must be aware of that**.

Interviewer: ‘Yeah …’

‘Since then, **I've learned to be more careful** with my everyday activity. I didn't realized how much mental things also have an influence on my available resources … and **I consider it more** when I'm planning my daily resources. My condition is more stable now’. (Hannah)

Some participants did not find the information all that useful, as they had already searched for it on the internet. Specific themes no longer meaningful for oneself were not seen as useful for oneself, although maybe useful for others:

There **was quite a lot of this kind of talk about what kinds of feelings this illness like has aroused**. And to be honest, it didn't seem so terribly rewarding precisely because they hadn't been thought about even then, back then like in the early stages of getting sick … In other words, they are by no means unnecessary, but think about them when the timing is right for you. (Arden)

#### 3.1.2 Workbook

In the intervention, information, tasks, exercises, and themes for group meetings were compiled in a *workbook*. The workbook helped participants to become aware of their own maladaptive thoughts and high standards and to build a new plan to change their mode of behaviour in an adaptive direction:

**With** the help of the **workbook**, **I noticed** that there is **no sense in my own thinking** … not at the beginning… not any realistic thoughts about my own recovery. **Too high goals** again.

Interviewer: ‘Yeah’.

So, **I edited** those goal stairs, and **I got it better** now.

Interviewer: ‘Okay, great!’

‘**Now I clearly regulate my energy** in situations, where I can save energy. And with exercise, I go to the gym 3 times a week, for 20 minutes. I always turn on the timer and then when it starts beeping, I stop. It feels good to have succeeded in that’. (Hannah)

The condition of two participants was classified as moderate to severe and their cognitive functioning was less fluent that of the other participants. They reported being unable to optimally benefit from or integrate information or their experiences.

### 3.2 Peer support

#### 3.2.1 Getting hope and perspective from other sufferers

One general goal for all participants was meeting peers and hearing about their way of managing. Meeting peers was one of the most valuable aspects of the intervention. Listening to peers' experiences activated participants to see their own situation from a different perspective and created hope for the future:

Seeing peers brought that **perspective** on myself that I have things pretty good as they are now. And that everyone has their own challenges. Also… you must find your tools by yourself. In a group, **through others I reflected on my situation** like looking at myself from the outside and **became more aware of my own situation, especially my strengths**. (Tina)

My peers gave me, most of all, **hope**, when I saw there were so many already getting part‐time work and all that. Maybe someday, I'll work part‐time, or at least, be more balanced in my daily activities. (Bellamy)

It was also important give others hope:

I noticed that my situation is now better than others'. So I hope, that I had a chance to **give hope** to others. It has been a long road from me, but getting in better shape, yes, it is possible. There is **hope**. (Tina)

While peer support was mostly seen as an advantage, a way of gaining hope from others, it was also seen as a double‐edged sword. One participant said that peer support awakened one's own fears of one's functioning getting worse:

**I haven't thought before** about **how worse** it can get. And how I'm gonna deal with it if my condition gets worse. (Alva)

#### 3.2.2 Shared experiences

Participants reported that sharing experiences of their illness was meaningful. Some had frequently experienced stigma and disparagement because of their illness, especially in encounters with healthcare specialists. Dealing with these experiences was meaningful and helped them to adjust and leave behind difficult feelings caused by such encounters:

I was so **relieved** to hear about peers' encounters with the healthcare system. That feeling is impossible to describe. But we **were all in the same boat**. That **relieved my feelings and my shame**, that this is in my head and my fault. With this realization of a **shared experience** I was somehow **ready to let go of these feelings some way, adjust and… focus better on life right now**. (Jessie)

#### 3.2.3 Practical tools from others

The most frequent peer category was receiving practical tools from others. The participants' goal was to find tools to manage in everyday life, and peers offered many practical tools that could help them cope with ME/CFS. Interestingly, tips from peers were more welcomed and differently experienced than tips from healthcare specialists:

A peer said think of the illness as white handbag you carry with you. You must carry it with you, but it is not you and you can leave it nearby you. So, if the doctor would had said that to me, **it would have sounded like a really belittling** thing, like a joke in a way. But when **a peer says it then it can be taken as a good tip**. I did that. Now it's little bit lighter to live with this condition. (Jenny)

*T*he experienced specialist was beneficial … **following her example**, it was easier to model my own behaviour in practice **… I was able to** go blueberry‐picking in the forest. I was picking for 15 minutes, then resting and later going back into the forest again**. It made me feel that, yes, I can still do things I enjoy**, and I need to practise it more. (Bellamy)

### 3.3 Shared guidance in personal processes

Although a group intervention is a process with a structured guide book and peer support, it is also an intra‐individual process that starts with defining its goals, after which it proceeds to its end in interaction with others. Experiencing the safe solving of problems together emphasis the value of the resources offered by shared guidance and growing self‐awareness as an outcome in the personal process.

#### 3.3.1 Experiencing the safe solving of problems together

In the individual process, safety was considered an important factor for going forward. Participants reported that safety was created by healthcare professionals who showed an interest, took the participant seriously, dispensed information, and most of all supported their individual needs by processing things together with them.

For me, it was important that **you heard my wishes**. It wasn't like that almost all the time someone says what you need to do. We think about the **problems and solutions together**. In a concrete way, this was seen in goal setting. The goals came from me **but you helped me** to make it concrete. (Hannah)

I felt that you succeeded in bringing a good group **together**, I meant that it felt a **psychologically safe space** for all of us …

Interviewer: ‘Yes’.

…that was seen, everybody speaks so freely and I believe it was a meaningful experience for all of us, because healthcare encounters are often not that **safe**. (Tina)

One participant also reported an experience in the group that did not feel safe and shared. It turned out that earlier experiences had influenced this reaction:

One thing, I'm going to say it straight now. I felt, I was psyched, we were doing breathing exercises. Just breathe and do exercises, and puff…you are cured! That own experience a bit different, breathing is not a miracle cure. It didn't feel that were in this **together**.

Interviewer: ‘Oh, I'm so sorry to hear that… Can you be specific; I want to understand what happened …’

I was going to say … I have had so much stigma on my way … **I've noticed that** when someone says just breathe **it is huge trigger** for me. It always feels that yeah, here we go again, there is nothing wrong with you and you are just imagining all this. (Mary)

#### 3.3.2 Growing self‐awareness

All the participants reported development in their self‐awareness, meaning better understanding of their thoughts, emotions, body signals and experiences and taking this into account in their actions. Better self‐awareness makes improvement possible.

**You helped me** to see me more closely how my twisted thoughts guide my actions. **I found myself** wondering in the same kinds of situations that is my thought here adequate or is it still leading me to destruction? I noticed, my thought said—you should do more—which was a trap! And I avoided it! This is still difficult but **now more aware of it**. (Tina)

The result was that I became **more aware of my actions**. It is difficult say just one thing, it's the growth inside the group. But with **your support** and the peers comments. Priceless. (Meredith)

Growing self‐awareness was also seen in changed values. Participants reported changes in their own thinking and their personal values:

I just realised that ever since I was a child, I have been a career person with high standards, but now I have realized that in this state, those **values** don't matter anymore. It's more important to find something to enjoy and go for it. (Mary)

This **process** and discussions with you have also helped me seen my life differently…

Interviewer: How?

I have noticed that **my worth** as a human has been tied to how I perform things, it's not healthy, and I now realise that. I think I need more help to understand my distorted thoughts (Anna)

## 4 DISCUSSION

This study explored participants' experiences of the usefulness and harmfulness of a psychological group intervention for patients with ME/CFS. Participants' set their personal goals for the intervention on shared guidance with a professional. The most wished‐for goals were peer support and learning pacing and ways to relax. Goals were mostly achieved. Participants reported that much remained for them to do in managing their illness themselves, and that after the intervention finding new goals for future management was much easier.

None of the participants reported experiencing any harm from the intervention. Overall, they experienced the intervention as useful. In some cases, when the themes for discussion were already familiar or their own functioning restricted their ability to learn, the intervention was seen as less useful. Participants found the intervention useful as a way of receiving information about their illness, peer support and shared guidance in their personal process.

While the structured protocol together with its contents was experienced as useful, the participants emphasised the benefits of the interactional processes, especially peer support. Practical tips from peers were welcome and helpful. Support and shared guidance in personal processes was the most frequently mentioned category: participants highlighted the importance of receiving support and guidance from professionals and the value of interaction based on working together and trying to find a shared understanding and solutions.

Participants reported that all the main factors—information, shared guidance, and peer support—interacted with each other, resulting in the activation of their own personal processes for going forward in their illness management and adjustment.

### 4.1 Structured protocols combined with interpersonal factors facilitated change

CBT‐based structured models focus on identifying negative thoughts, practising new skills, goal setting, problem solving and self‐monitoring.^4^ In this study, the participants found the structured, CBT‐based tailor‐made intervention, with themes commonly used in earlier CBT models, such as identifying harmful thoughts and self‐monitoring, useful. To improve adjustment and illness management with ME/CFS requires opportunities for patients to practice health‐related behaviour, strengthen their functional capacity and maintain their motivation to continue with their illness management and adjustment. Hence, CBT‐based tailor‐made interventions for ME/CFS patients are useful.

Participants reported on the therapeutic role—shared guidance—as a meaningful and influential factor in personal adjustment and illness management. They also highlighted their feelings of psychological safety, a novel finding. Theoretically, the importance of psychological safety is recognised in organisational and psychotherapy research as a multidimensional, dynamic phenomenon that describes the perceived safety of taking interpersonal risks.^36^ It promotes individuals' voices, learning behaviour, and support of and familiarity with others.^37^, ^38^ Interpersonal relations influence the outcome of psychotherapy, the starting point of which is the feeling of being safe.^39^ Safety is also created by healthcare providers when aiming to counterbalance and validate their patients' experience and understanding of their condition and encourage them to manage their illness using their own agency.^31^

The participants stressed the role of peers and their influence in better illness management. Earlier studies have also noted the need and benefit of social support in coping with illness. The study by Moncorps et al.^30^ concluded that it would be worth exploring the incorporation of patients' experiential knowledge in intervention development and research, especially in relation to ME/CFS. A better understanding of different coping strategies would help health promotion researchers, managers, and clinicians, as also found in our study.

### 4.2 Clinical implications

This study identified three different themes that should be stressed in the clinical work with ME/CFS patients. First, a health‐behavioural approach is needed in designing interventions for patients with ME/CFS. Second, peer support should be considered as a resource and implemented together with healthcare experts as it has many benefits, such as better activating individual processes, validating harmful experiences and providing practical tools for patients in their everyday lives. Third, encounters with health care practitioners that are meaningful and include safe guidance and collaborative problem solving create possibilities for better illness management and adaptation to life with ME/CFS. A lot of stigma continues to surround ME/CFS. Shared guidance in planning one's own rehabilitation without fear of being stigmatised is crucial and is supported by current guidelines.^4^ Our results are in line with those of Clark and Holttum^11^ and Hareide et al.,^31^ who emphasise the importance of patients feeling safe and understood in their medical encounters.

### 4.3 Study limitations and directions for future research

As is typical in qualitative research, this study was conducted with only a small number of participants^35^ and did not to attempt to represent the experience of all ME/CFS patients. Thus, the results are not generalisable to all ME/CFS patients. Moreover, participation was voluntary. The results might have been different had the study included participants with a less positive image of CBT‐based interventions. Further, as the first author was responsible for collecting the data and delivering the intervention, social desirability bias should be evaluated.^35^ The fact that the first author got to know the participants during the intervention could have influenced the participants' feedback, through their wanting to please the author and say what they think the author wanted to hear.^35^ On the other hand, gaining familiarity with the participants may also have enabled them to be more honest in their answers, as shown in the results, where one participant reported not being understood.

Future research could usefully study ME/CFS patients discursive choices on illness management and the restructuring of identity. As Hareide et al.^31^ also suggest, the role of the therapeutic alliance and therapeutic processes and/or the role of an experienced specialist in treating patients with ME/CFS could also be further studied. Longitudinal qualitative studies could also supplement quantitative research data to design interventions that better meet patients' needs.

### 4.4 Conclusion

The results of this study underline the importance of developing interventions for ME/CFS that include the wishes, needs and experiences of sufferers. The present health psychological group intervention was viewed by the participants as an acceptable and wished‐for experience. Interactional processes with peer support and health care experts should be given greater consideration as influential factors for achieving better illness management and adjustment and should also be further researched and utilised more in developing interventions for patients with ME/CFS.

## AUTHOR CONTRIBUTIONS

Meeri Keurulainen, Elina Wallenius, Mikko Pänkäläinen, Jukka Hintikka and Markku Partinen prepared the preliminary research plan. Meeri Keurulainen, Elina Wallenius and Mikko Pänkäläinen collected the material, which Juha Holma and Meeri Keurulainen analysed qualitatively. Meeri Keurulainen wrote the first version of the manuscript, in the finalisation of which other authors have contributed significantly. All authors have read and approved the final version of the manuscript.

## CONFLICT OF INTEREST STATEMENT

The authors declare no conflict of interest.

## ETHICS STATEMENT

Participants have not provided consent for full transcripts to be made available outside this study.

## Supporting information

Supporting information.

## Data Availability

Research data are not shared.
