# Supplementary material for: ‘I became more aware of my actions’—A qualitative longitudinal study of a health psychological group intervention for patients with myalgic encephalomyelitis/chronic fatigue syndrome
Source: Health Expect. 2023 Aug 1;26(6):2312–24. doi: 10.1111/hex.13833 (PMC10632634; doi:10.1111/hex.13833)
Supplement: Supplementary file 1 — Supporting information. [file HEX-26--s001.docx]

**Interview before the intervention**

1*. What information have you received about ME//CFS?* Where did you find the information? Is it possible to recover from chronic fatigue syndrome or try to improve functional capacity? If so, how?

2. *What* *was your life like before ME/CFS?* What did you do and what did you enjoy?

3. *How has the illness affected your life?* Your ability to function? Your self- image? Your relationships?

4. *Describe a little about your current everyday life, what is it like?* Do you work? Can you meet people? Everyday activities? Moving, standing up, lying down?

5. *What is the most difficult thing for you in everyday life?* What do you need help with? What things could help you manage better than before?

6. *What ways and means have you developed to cope in everyday life?*

*7. How do you feel about working in a group?* How do you react if different opinions or different ways of dealing with the illness come up in the group?

*8. How does stress affect your symptoms and coping? What things stress you out?* How do you relieve stress?

*9. How do you feel about setbacks?* Do they make you feel discouraged, angry and try twice as hard? To surrender?

*10. What do you hope and expect from the rehabilitation period? Could you name two or three rehabilita*tion goals?

*11. What are your strengths in terms of rehabilitation, what you are good at? What about challenges, is there any issue you have often failed in?*

*12. What do you think about your future? Where will you be in 5 years’ time?*

**Final interview and 3-month follow-up interview**

1**. Description of present state after the end of the group intervention, change from the beginning of the group intervention:**

*How are you doing in everyday life/work at the moment?*

*Have you received help or support during the study/intervention from somewhere else?*

2. **Experiences** **of the rehabilitation period:**

*What kind of experience was the group rehabilitation?*

*Were there things which felt good or conducive to your own rehabilitation? If so, can you tell me in more detail in what way?*

*What didn't feel good or workable for you? If so, can you tell me in more detail in what way?*

*Was the rehabilitation harmful? If so, can you tell me in more detail in what way?*

*What would you change/add to the rehabilitation if you could decide for yourself?*

*What aspects of your own rehabilitation should you focus on/pay attention to in the future?*

3. **Expectations and goals for rehabilitation and orientation to the future:**

*Were your expectations of the rehabilitation period fulfilled? If so, can you tell me about them more specifically?*

*What goals didn`t you achieve and why?*

*What do you think about your future right now? Where will you be in five years?*
